# Expansion of an Academic Molecular Tumor Board to Enhance Access to Biomarker-Driven Trials and Therapies in the Rural Southeastern United States

**DOI:** 10.3390/curroncol31110534

**Published:** 2024-11-16

**Authors:** Anivarya Kumar, Jennifer R. Owen, Nicholette T. Sloat, Elizabeth Maynard, Vanessa M. Hill, Christopher B. Hubbard, Matthew S. McKinney, Linda M. Sutton, Shannon J. McCall, Michael B. Datto, Ashley N. Moyer, Bennett A. Caughey, John H. Strickler, Ryne C. Ramaker

**Affiliations:** 1 Duke University School of Medicine, Durham, NC 27710, USA; anivarya.kumar@duke.edu; 2 Duke Cancer Network, Durham, NC 27707, USAvanessa.hill@duke.edu (V.M.H.); linda.sutton@duke.edu (L.M.S.); 3 Duke Cancer Institute, Durham, NC 27710, USAmatthew.mckinney@dm.duke.edu (M.S.M.);; 4 Department of Pathology, Duke University School of Medicine, Durham, NC 27710, USA; chris.hubbard@duke.edu (C.B.H.); shannon.mccall@duke.edu (S.J.M.); michael.datto@duke.edu (M.B.D.); ashley.n.moyer@duke.edu (A.N.M.); 5 Division of Hematologic Malignancies and Cellular Therapy, Department of Medicine, Duke University, Duke University Medical Center, Durham, NC 27710, USA; 6 Division of Hematology/Oncology, Massachusetts General Hospital, Boston, MA 02114, USA; bcaughey1@mgb.org; 7 Division of Medical Oncology, Department of Medicine, Duke University Medical Center, Durham, NC 27710, USA

**Keywords:** cancer disparities, targeted therapy, molecular profiling, comprehensive genomic profiling

## Abstract

Targeting tumor-specific molecular alterations has shown significant clinical benefit. Molecular tumor boards (MTBs) connect cancer patients with personalized treatments and clinical trials. However, rural cancer centers often have limited access to MTB expertise. We established an academic–community partnership expanding our academic MTB to affiliated rural community cancer centers. We developed a centralized molecular registry of tumors (MRT) to aggregate the comprehensive genomic profiling (CGP) results and facilitate multidisciplinary MTB review. Of the 151 patients included, 87 (58%) had actionable genomic biomarkers, 42 (28%) were eligible for a targeted off-label therapy, and 27 (18%) were matched to a clinical trial. Of those with a clinical trial match, only 1 of 27 (3%) was enrolled in the identified trial. One year into implementation, community oncology providers were anonymously surveyed on persistent barriers to precision treatment utilization. The primary barriers to clinical trial enrollment were the distance to the trial center (70%), lack of transportation (55%), and lack of local trials (50%). This study offers a framework to improve access to molecular expertise, but significant barriers to the equitable use of CGP and trial enrollment persist.

## 1. Introduction

Precision oncology, or the use of comprehensive genomic profiling (CGP) to identify and target actionable biomarkers, has revolutionized cancer treatment by improving outcomes in patients with solid and hematologic malignancies [1]. Increasingly, genomic biomarkers are a critical selection criterion in clinical trials. This individualized approach, with associated clinical trials and targeted therapies, has improved both the longevity and quality of life for patients and has increasingly become the standard of care [2,3]. However, historically underserved patient populations undergo molecular testing less frequently and have less access to targeted therapies and clinical trials than those receiving care at urban hospitals or academic centers [4,5]. This disparity in access to molecular testing and treatments negatively impacts health outcomes for patients in rural communities, particularly those communities with high rates of racial and ethnic minorities, low socioeconomic status, and limited health literacy [6].

A key driver of this disparity is the lack of access to the clinical decision-making and trial-matching support that a molecular tumor board (MTB) provides [7]. MTBs assemble a team of medical oncologists, pathologists, molecular scientists, clinical trial staff, and genetic counselors who interpret tumor genomic data for actionable biomarkers and match patients to corresponding targeted therapies and trials. Unfortunately, MTB support is often limited to academic or high-volume community oncology centers, and oncologists practicing in rural communities are less likely to order CGP due to a lack of timely access to multi-marker panels and personnel to help interpret test results [8]. Further, studies show that when patients and oncology clinicians in rural and urban communities are given uniform access to targeted clinical trials, the gap in patient survival outcomes closes, suggesting that prioritizing precision oncology services for otherwise marginalized patients may be important to providing equitable cancer outcomes [9]. While the extension of MTB support to the community shows great promise in advancing equity in cancer care, reports of successfully integrated academic-community MTB partnerships are limited [10,11].

Here, we present data from an academic-community MTB partnership in which the Duke Cancer Institute (DCI), a National Cancer Institute (NCI)-designated comprehensive cancer center and member institution of National Comprehensive Cancer Network (NCCN), established an information technology and regulatory structure for the expansion of MTB support to the Duke Cancer Network (DCN), a series of affiliated rural community cancer centers across North Carolina. In this manuscript, we describe our experience implementing this partnership and highlight the remaining barriers to patient care, as reported by oncologists serving these communities.

## 2. Materials and Methods

### 2.1. Study Population

We consented and enrolled 151 patients from five affiliated community oncology sites for review by the Duke MTB between 1 January 2022 and 31 January 2024. The five community sites included were spread across rural areas across the state of North Carolina in the cities of Lumberton, Clayton, Smithfield, Henderson, and Laurinburg. Patient data were collected under an IRB-approved protocol. The Duke University Health System Institutional Review Board (DUHS IRB) and Local Review Committees at each individual site approved the study, and all patients provided verbal or written informed consent prior to participation. Eligibility criteria included age 18 years or older, a cancer diagnosis, and status as an active patient of one of the participating sites. Use of Duke infrastructure for clinical trial matching was approved by the DUHS IRB with a waiver of informed consent under an existing protocol.

### 2.2. Molecular Tumor Registry Workflow

A regulatory and technological infrastructure was previously developed at DCI for centralized storage and review of molecular data as well as clinical trial matching, as previously described [12]. Briefly, data sharing agreements were established with commercial vendors to directly deposit structured molecular data into a centralized data warehouse, the Duke Molecular Registry of Tumors (MRT). These data are normalized across vendor platforms to facilitate review.

MRT uses an SQL database to generate tables for variants, variant types, pathogenic markers, biomarkers, transcripts, chromosomes, genes, body sites, diagnoses, clinical events, orderable test names, trials, and other data elements used to define genomic results. In addition, patients are molecularly matched to clinical trials open at Duke. Clinicians participating in the MTB access MRT-based therapy and trial matches through a secure web-based portal. Additional clinical characteristics of patient demographics, treatment history, and disease status are imported from electronic medical record (EMR) chart reviews.

The MTB’s program coordinator, medical oncology co-leaders, molecular pathologist, clinical trial staff, and a genetic counselor review CGP results stored in MRT from the previous week and discuss actionable findings, atypical or unexpected results, potential germline implications, or make the patient eligible for biomarker-driven clinical trials. Multidisciplinary MTB meetings are held weekly to discuss selected cases. Discussion notes are recorded and sent to the ordering provider through secure e-mail within 24 h.

In order to extend this platform to participating community sites, a new regulatory framework was developed. Data sharing agreements were established between individual participating hospitals, commercial vendors, and the primary coordinating academic center. Individual patient consent enabled the direct delivery of structured molecular data into an electronic instance of MRT that was siloed for the participating sites in aggregate and made relevant clinical data available to central MTB staff. Once consent was obtained and the data delivered, patient CGP reports were reviewed at the MTB within 1 week and recommendations were returned to the treating clinician. All participating community site providers ordered CGP tests performed by Clinical Laboratory Improvement Amendments (CLIA)-accredited vendors. For the purposes of this study, participating vendors were Foundation Medicine (Cambridge, MA, USA) and Guardant Health (Palo Alto, CA, USA).

### 2.3. Provider-Reported Barriers to CGP and Targeted Therapy

One year into implementation, participating community site providers were surveyed on attitudes and barriers associated with accessing CGP and implementing the recommended trial or therapy (Appendix A). This portion of the study was granted an exemption by the DUHS IRB. Survey questions and structure can be found in the Appendix A. Responses were anonymous and stored on REDCap for analysis (Research Electronic Data Capture, Vanderbilt, TN, USA) [13].

## 3. Results

### 3.1. Characteristics of Patients Enrolled

**Figure 1:**
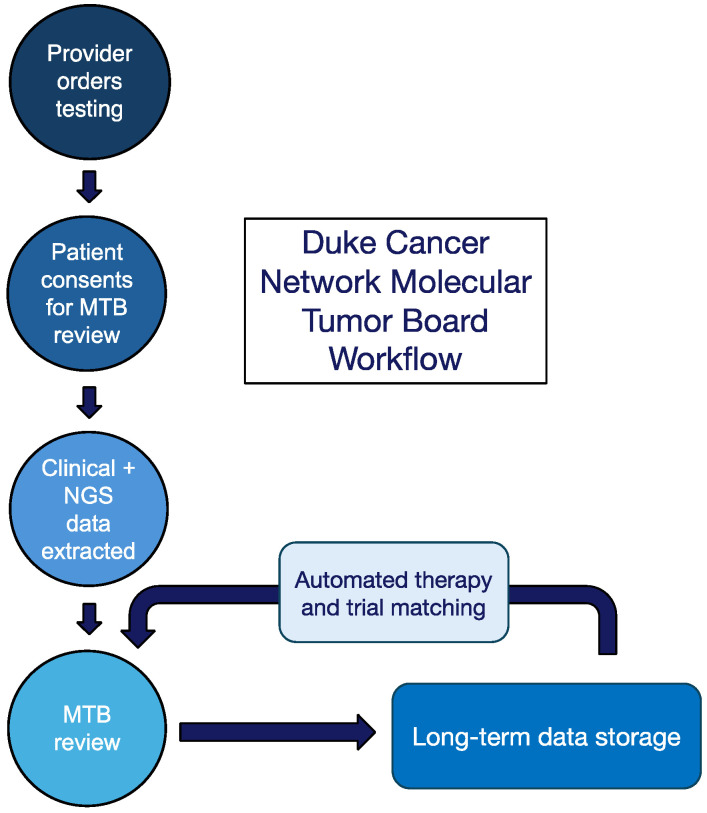
Flow diagram describing the workflow of Duke MTB review for community centers.

**Figure 2:**
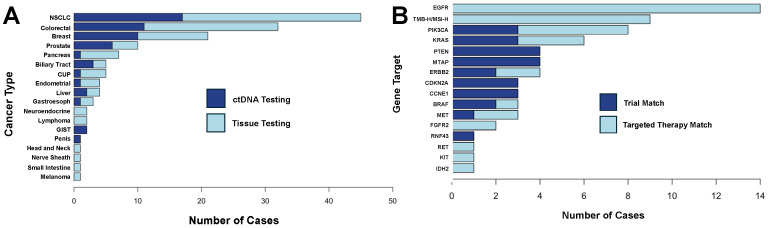
Cancer types and molecular targets reviewed. (**A**) Bar chart detailing the cancer types reviewed for this study. Colors represent the number of cases profiled by circulating tumor DNA (ctDNA) or tissue sequencing. (**B**) Bar chart showing the genes identified with a clinical trial or targeted therapy. Colors represent the number of cases for which either a clinical trial or FDA-approved targeted therapy was available. If both a targeted therapy and a clinical trial was matched (*N* = 9), the patients were colored based on their clinical trial match.

A total of 151 DCN patients completed the Duke MTB workflow (Figure 1). Our patient population was diverse, with 45 (30%) self-identifying as Black or African American, 35 (23%) as Native American or American Indian, and 5 (3%) as Hispanic or Latino ethnicity. Most patients (121, 80%) had public insurance with Medicaid and/or Medicare and five (3%) had no insurance coverage. (Table 1). A wide variety of tumor types was represented: non-small cell lung cancer (30%), colorectal cancer (21%), breast cancer (14%), prostate cancer (7%), and other types of cancer (28%). A total of 93 patients underwent tissue profiling and 58 received blood-based profiling across 18 cancer types (Figure 2A).

### 3.2. Actionable Biomarkers Found for Patients

The majority of patients (58%) were found to have at least one biomarker predicting response to therapy, matching clinical trial inclusion criteria, or requiring follow-up germline testing. We found 33 (22%) patients with a biomarker linked to an FDA-approved therapy, 18 (12%) patients met molecular criteria for an open clinical trial at DCI, and 9 (6%) patients had both a targeted therapy and clinical trial match (Figure 2B). Additional findings included 20 (13%) patients with a biomarker associated with therapeutic resistance, 9 (6%) patients with a biomarker associated with an off-label therapy, and 15 (10%) patients with findings indicating a referral to medical genetics. For patients with an FDA-approved or off-label therapy match, 19 (45%) were already being treated with the identified therapy, 7 (17%) were subsequently started on the identified therapy, and 11 (26%) were deceased prior to the end of our data cutoff. Of the 27 patients found to have a potential clinical trial match, only 1 (3%) was successfully enrolled in a clinical trial (Table 2).

The most common biomarker-associated therapy recommendation (nine cases) was the use of anti-EGFR therapy for patients with left-sided colon or rectal cancer in the absence of a contraindicating RAS/BRAF mutation (Table 3) [14]. Of the nine cases identified, only two patients were receiving guideline-directed anti-EGFR therapy, highlighting this patient population as one with potential for increased targeted therapy use. After our recommendations, two patients were immediately started on anti-EGFR therapy. Recommendations for immune checkpoint inhibitor therapy, either as a single agent (seven cases) or in combination (two cases), were provided for patients with microsatellite instability (MSI-H) or high tumor mutation burden (TMB-H) across a variety of tumor types [15,16]. The vast majority (seven out of nine) of these patients had already been identified as appropriate candidates for checkpoint inhibitor therapy. Use of apelisib or capivasertib for PIK3CA-mutated breast cancer was the third most common target identified by our MTB and all patients had either already received targeted therapy or started this therapy shortly after our recommendation [17,18].

In addition to these frequently observed biomarker targets, we identified several cases with rare or unexpected CGP results. One individual with metastatic, chemotherapy-refractory squamous cell carcinoma of the maxillary sinus was found to have an EGFR exon 20 insertion. EGFR mutations are rare in head and neck cancer, although they have been described in small cohorts as a potential therapeutic target [19]. A patient with metastatic breast cancer was found to have an activating BRAF^V600E^ mutation, which has only previously been described in case reports. In several of these reports, partial responses to combination BRAF and MEK inhibitor therapy has been observed [20,21]. An activating RET fusion was identified in a patient with resected colon cancer. In large cohort studies, RET fusions have been identified in less than 1% of colorectal cancer cases [22]. In each of these cases, the patient had not received the associated targeted therapy at the time of data collection. Longitudinal follow-up of these actionable findings is ongoing.

### 3.3. Community Oncology Provider Survey Results

**Figure 3:**
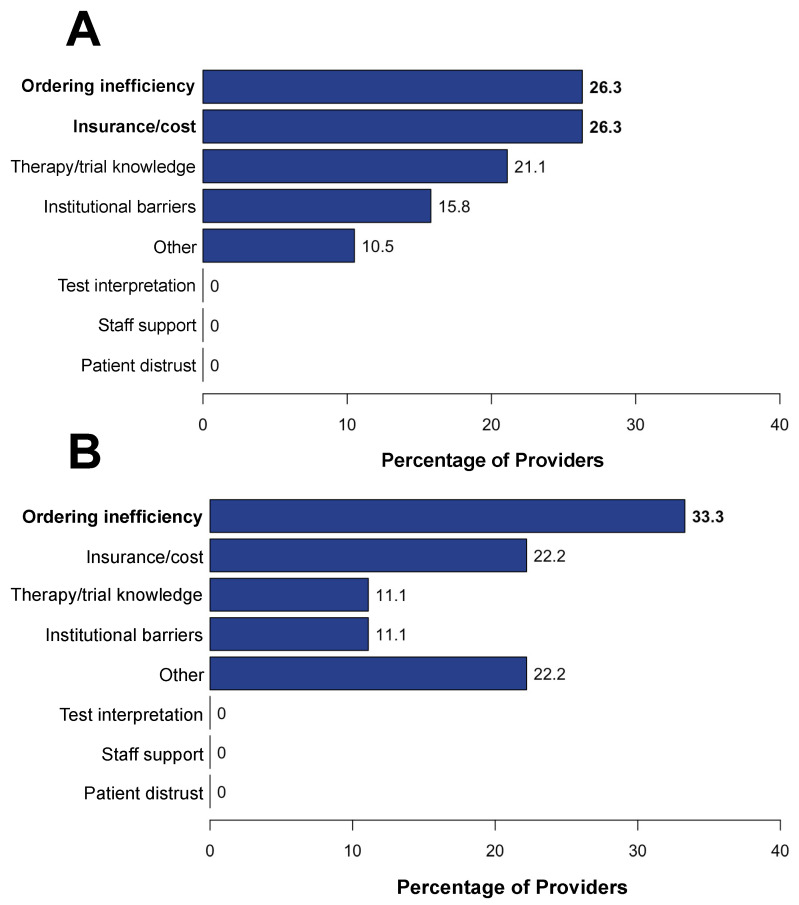
Barriers to CGP. (**A**) Bar chart with provider-reported barriers to ordering CGP. (**B**) Bar chart with provider-reported barriers to completing CGP after ordering. The “Other” bar includes the following responses: difficulty choosing a molecular testing vendor.

**Figure 4:**
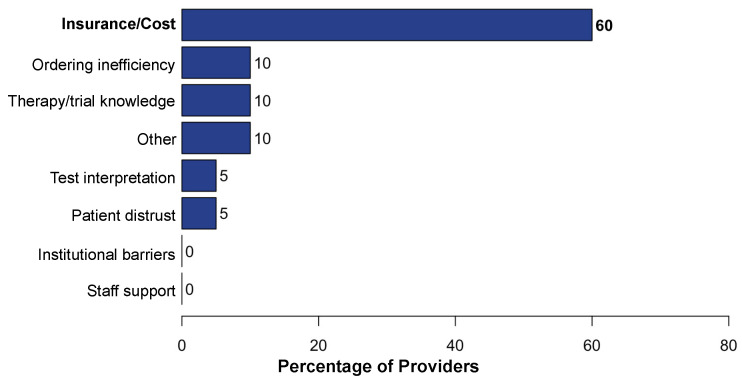
Bar chart describing provider-reported barriers to use of biomarker-associated targeted therapy. The “Other” bar includes the following responses: inadequate specimen size (*N* = 1), limited experience with administering targeted therapy (*N* = 1).

**Figure 5:**
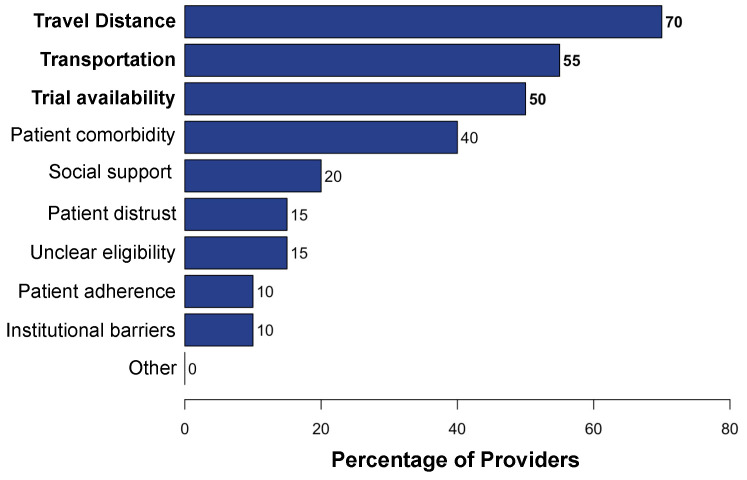
Bar chart showing provider-reported barriers to patient clinical trial enrollment.

Among 26 participating community site providers, 20 (77%) completed the survey on barriers to accessing CGP, targeted therapies, and clinical trials. These providers included attendings, hematology–oncology fellows, and advanced practice providers. Our survey explored barriers at each point in management including CGP ordering, test completion and receipt of results, and the initiation of targeted therapy. While 18 respondents (90%) reported that molecular testing is very important to their patients’ care, only 9 respondents (45%) reported that they were comfortable interpreting results on their own. The primary challenges to ordering CGP was patient cost and/or insurance status as well as difficulty navigating the ordering process (26%; Figure 3A), and the primary challenge to completing CGP was difficulty navigating the ordering process (33%; Figure 3B). From the time of matching a biomarker-driven targeted therapy to receipt of the targeted therapy, the primary barrier reported was patient cost and/or insurance (60%; Figure 4). Across the provider responses to questions addressing barriers ordering CGP, completing CGP, and receiving CGP-based therapy, a relatively high percentage chose “other” and described difficulty choosing which molecular testing company to use when placing an order, having inadequate tumor specimen for test completion, and having limited experience administering specific targeted therapies. Multiple barriers to enrollment in targeted therapy clinical trials were identified. These included the distance to the closest trial site (70%), lack of transportation (55%), and lack of available local trials (50%; Figure 5).

## 4. Discussion

This study demonstrated that centralized review by an academic MTB to support community cancer centers servicing rural and traditionally underserved patients is feasible and may improve patient care and access. Our review of over 150 cases expands upon and validates previous studies that developed academic and community center partnerships around MTBs [11,23,24,25]. The DCI’s MTB expansion to rural oncology sites across the southeastern United States enabled the identification of actionable biomarkers and clinical trial eligibility for patients that otherwise may not have had access. Using our genomic database and regulatory framework, we were able to aggregate genomic profiling results from multiple vendors at both academic and community centers and use these data as a first step to overcome barriers to guideline-based care for underserved cancer patients. Our MTB model is both sustainable and scalable, serving as a template for the regulatory and technical development of MTB partnerships between academic cancer centers and community oncology sites nationally.

While the value of MTB expansion is demonstrated by the high proportion of actionable biomarkers, provider-based surveys suggest that academic MTB support may also encourage a greater utilization of CGP. The finding that almost all oncologists view CGP as critical to their patients’ care but less than half feel comfortable interpreting the results indicates an unmet need in provider support. MTBs fill an important role by equipping medical oncologists with the tools to offer guideline-recommended treatment. Freedman et al. 2018 reported that while 85% of oncologists with access to an MTB used next-generation sequencing (NGS), only 71% of oncologists without access to an MTB were able to use NGS to guide their patients’ treatment plan, further supporting partnership with academic MTBs as a tool for optimizing care delivery [26].

While an appreciable number of patients were matched to and received an FDA-approved therapy, roughly one-third of these patients never received their matched therapy, suggesting a need for improvement. Also, only one patient was enrolled in a clinical trial despite a significant number of potential trial matches. This finding aligns with previously reported low clinical trial participation rates [11]. Bruno et al. 2022 reported clinical trial enrollment as low as 3.9% in White patients and 2.1% in Black patients across multiple cancer types [27]. Many studies have proposed factors that contribute to low rates of targeted therapy use and clinical trial participation. These factors include systemic barriers such as unaffordable cost, long waiting lists for treatment, and the frequency of study visits as well as patient barriers including fear of side effects, discomfort with random assignment to control, and lack of financial reimbursement for travel to treatment centers [28,29,30,31,32]. Various patient demographics have also been associated with underrepresentation in clinical therapies and trials including racial and ethnic minorities, older age, low socioeconomic status, and limited English proficiency [33,34,35,36]. The interrelation of these factors makes it difficult to parse which barriers are most salient. There are a number of steps from molecular testing to the appropriate delivery of targeted therapy treatment and it remains a challenge to delineate actionable barriers at each stage of the process. While prior studies have identified clinical practice gaps from reviews of annotated patient databases, this study provides clinician-reported barriers at each stage of the precision oncology process—performing CGP, receiving the CGP interpretation, ordering targeted therapy, enrolling in a clinical trial—to pinpoint and address the most critical barriers implementing precision oncology based on the personal experience of providers working in a community health setting [37].

The findings from our survey are two-fold. First, the primary social barriers to receiving targeted therapy are the cost of treatment and limited health literacy around treatment options. The barriers for enrolling in a clinical trial are centered around the geographic location. This aligns with the cost burden analyses of targeted anticancer medicines for low-income patients, studies of low health literacy impacting cancer decision-making, and reports of clinical trials having poor geographic accessibility [38,39,40,41,42]. Second, while MTB expansion can address some barriers to ordering CGP and matching patients with targeted treatments for more favorable cancer outcomes, it has not addressed the remaining barriers such as the cost of care and travel burden to clinical trial sites. Additionally, there may be disparities not readily apparent in our data as only patients who survived long enough to undergo CGP and consented to inclusion were enrolled. Although academic–community MTB partnerships take important first steps in improving access to equitable treatment options for rural and urban cancer populations, community initiatives addressing financial toxicity, health literacy, and geographic isolation remain a priority for resolving disparities in precision oncology.

Our findings serve as a foundation for the development of strategies enabling equitable precision treatment by specifically targeting cost, health literacy, and geographic barriers in rural counties. Potential avenues for improving access to targeted therapy include provider education on cost-conscious clinical pathways, drug assistance programs for financial coverage options, and grant programs that help offset cancer-related expenses [43]. Coupled with more efficient testing strategies, further initiatives for the more equitable delivery of targeted therapy delivery include patient education on treatment options and plans, provider training on the simplification of complex cancer jargon and “teach-back” methodologies, and institutional collaborations with precision medicine companies to create patient-friendly material on the molecular testing process [44]. Geospatial determinants of health are a critical target for improving clinical trial enrollment. Given the high proportion of providers identifying distance and a lack of transportation to trial sites as the primary barrier to trial accrual, multilevel interventions such as the establishment of robust non-emergency medical transportation (NEMT) programs, collaboration with rideshare platforms, and application for transportation service grants could be prioritized at cancer centers with active clinical trials [45,46].

## 5. Conclusions

As the repertoire of targeted cancer therapies continues to expand, the need for a scalable framework to deliver targeted therapy to historically underrepresented patients will continue to grow. Here, we demonstrate that the expansion of an academic MTB to affiliated community cancer centers is feasible. In conjunction with interventions addressing cancer-related costs, literacy, and transportation, this approach has the potential to increase the rates of precision oncology delivery to underserved communities and mitigate rural versus urban disparities in cancer outcomes.

## Figures and Tables

**Table 1:** Patient demographics.

Total Enrollment, *N*	151
Female, *N* (%)	74 (49.0)
Age, median (range)	68 (27–89)
Race, *N* (%)	
Black	45 (29.8)
Native American	35 (23.2)
White	65 (43.0)
Unknown	6 (4.0)
Ethnicity, *N* (%)	
Hispanic	5 (3.3)
Non-Hispanic	143 (94.7)
Unknown	3 (2.0)
Insurance Type, *N* (%)	
Private	26 (17.2)
Medicare	99 (65.6)
Medicaid	19 (12.6)
Veterans Affairs	2 (1.3)

**Table 2:** Biomarker results.

Biomarker Result	Patients Eligible, *N* (%)
FDA-approved therapy Received Therapy	33 (21.8) 26 (17.2)
Targeted clinical trial Enrolled in Trial	27 (17.9) 1 (0.7)
Off-label therapy	9 (6.0)
Received Therapy	2 (1.3)
Therapeutic resistance	20 (13.2)
Germline implications	15 (10.0)
None	64 (42.4)

**Table 3:** Biomarker-associated treatment patterns.

Biomarker Category	Patients Identified, *N* (%)	Already Receiving Therapy, *N* (%)	Started Therapy Post-MTB, *N* (%)	Deceased Prior to Initiation, *N* (%)
RAS/RAF WT colorectal cancer	9 (6.0)	2 (1.3)	2 (1.3)	4 (2.6)
TMB-H/MSI-H	9 (6.0)	7 (4.6)	0 (0.0)	0 (0.0)
PIK3CA-mutated breast cancer	5 (3.3)	3 (2.0)	2 (1.3)	0 (0.0)
KRAS G12C	3 (2.0)	1 (0.7)	0 (0.0)	0 (0.0)
EGFR exon 20 indel	3 (2.0)	0 (0.0)	1 (0.7)	2 (1.3)
MET-activating	2 (1.3)	0 (0.0)	0 (0.0)	0 (0.0)
FGFR2-activating	2 (1.3)	0 (0.0)	1 (0.7)	1 (0.7)
ERBB2 amplification	2 (1.3)	1 (0.7)	0 (0.0)	1 (0.7)
EGFR exon 19 or L858R	2 (1.3)	2 (1.3)	0 (0.0)	0 (0.0)
RET fusion	1 (0.7)	0 (0.0)	0 (0.0)	1 (0.7)
KIT-activating	1 (0.7)	1 (0.7)	0 (0.0)	0 (0.0)
IDH2-activating	1 (0.7)	0 (0.0)	1 (0.7)	0 (0.0)
BRAF V600E	1 (0.7)	0 (0.0)	0 (0.0)	0 (0.0)

## Data Availability

The data underlying this article are available in Appendix A or the figures and tables presented.

## Appendix A

### Survey Distributed to DCN Providers

The purpose of this survey is to identify barriers to appropriate use and application of molecular testing at DCN sites. Your answers will inform how to improve our processes to better support clinicians and patients ordering these tests. Please describe your role at the DCN below.

1. What is your role on the medical team?

1. MD attending
2. Advanced Practice Provider (PA or NP)
3. MD fellow
4. Other (specify)

2. At which DCN site(s) do you work?

3. What is the predominant type of cancer you see?

1. Thoracic
2. Gastrointestinal
3. Breast
4. Genitourinary
5. Malignant Hematology
6. Other (specify)

The Duke Molecular Tumor Board (MTB) launched an initiative in January 2022 to provide molecular interpretation and support services to the DCN, currently open at 3 sites.

1. Are you aware that the Duke MTB offers molecular interpretation and support services to the DCN?
  1. Yes
  2. No
  3. Unsure
2. Have you received an email or other communication from the Duke MTB providing an interpretation of a patient’s molecular test?
  1. Yes
  2. No
  3. Unsure
3. If so, were the interpretations useful?
  1. Yes
  2. No
  3. Unsure
4. Please explain, including any suggestions for improvement. [Insert free text]

Molecular testing includes single gene DNA tests such as: EGFR mutations in NSCLC; comprehensive multi-gene DNA-based panels offered by Foundation Medicine and Guardant; and RNA-based fusion panels.

1. How comfortable are you with ordering molecular testing for patients with cancer?
  - Very uncomfortable
  - Uncomfortable
  - Neutral
  - Comfortable
  - Very comfortable
2. How comfortable are you with interpreting molecular testing for patients with cancer?
  - Very uncomfortable
  - Uncomfortable
  - Neutral
  - Comfortable
  - Very comfortable
3. Molecular testing is important for the care of patients with cancer.
  - Very unimportant
  - Unimportant
  - Neutral
  - Important
  - Very important
4. Which molecular test do you order most often?
  - FoundationOne
  - FoundationOne liquid
  - Guardant360
  - Caris
  - Tempus
  - Internal test
  - Other (specify)

There are many barriers to the appropriate use of molecular testing in oncology; these may be especially acute for underserved populations.

1. How challenging is it to order molecular testing at your site?
  - Very difficult
  - Difficult
  - Neutral
  - Easy
  - Very easy
2. What is the greatest challenge to ordering molecular testing?
  - Insurance/cost
  - Institutional barriers
  - Patient distrust or skepticism
  - Ordering process is opaque or inefficient
  - Lack of knowledge or discomfort with interpretation
  - Lack of understanding of therapies/trials
  - Lack of staff support
  - Other (specify)
3. Once a molecular test has been ordered, how challenging is it to have the test completed?
  - Very difficult
  - Difficult
  - Neutral
  - Easy
  - Very easy
4. What is the greatest challenge to a molecular test being completed?
  - Insurance/cost
  - Institutional barriers
  - Patient distrust or skepticism
  - Ordering process is opaque or inefficient
  - Lack of knowledge or discomfort with interpretation
  - Lack of understanding of therapies/trials
  - Lack of staff support
  - Other (specify)
5. How challenging is it to order indicated targeted therapies for patients?
  - Very difficult
  - Difficult
  - Neutral
  - Easy
  - Very easy
6. What is the greatest challenge to ordering indicated targeted therapies for patients?
  - Insurance/cost
  - Institutional barriers
  - Patient distrust or skepticism
  - Ordering process is opaque or inefficient
  - Lack of knowledge or discomfort with interpretation
  - Lack of understanding of therapies/trials
  - Lack of staff support
  - Other (specify)

One of the main goals of the molecular testing and the DCN MTB initiative is connecting patients to clinical trials, when appropriate.

1. In your experience, how often do you have patients who you consider clinically appropriate for a molecularly matched clinical trial? For example, a patient with an actionable mutation.
  - Very rarely
  - Rarely
  - Somewhat often
  - Often
  - Very often
2. How often are you successful in referring patients for a trial?
  - Very difficult
  - Difficult
  - Neutral
  - Easy
  - Very easy
3. What challenges do you see as preventing patients from clinical trial enrollment? (select up to 3)
  - Patient distrust or skepticism
  - Patient medical co-morbidities
  - Poor adherence or active drug use
  - Distance to clinical trials
  - Lack of transportation
  - Lack of social support
  - Lack of available trials
  - Institutional barriers
  - I do not know when my patient is eligible
  - Other (specify)
